# Biological and clinical effects of abiraterone on anti-resorptive and anabolic activity in bone microenvironment

**DOI:** 10.18632/oncotarget.3724

**Published:** 2015-03-30

**Authors:** Michele Iuliani, Francesco Pantano, Consuelo Buttigliero, Marco Fioramonti, Valentina Bertaglia, Bruno Vincenzi, Alice Zoccoli, Giulia Ribelli, Marcello Tucci, Francesca Vignani, Alfredo Berruti, Giorgio Vittorio Scagliotti, Giuseppe Tonini, Daniele Santini

**Affiliations:** ^1^ Translational Oncology Laboratory, Medical Oncology, University Campus Bio-Medico of Rome, Rome, Italy; ^2^ Department of Oncology, University of Turin, San Luigi Hospital, Orbassano, Turin, Italy; ^3^ U.O. Oncologia Medica, Ospedali Civili di Brescia, Brescia, Italy

**Keywords:** abiraterone acetate, osteoclast, osteoblast, bone marker

## Abstract

Abiraterone acetate (ABI) is associated not only with a significant survival advantage in both chemotherapy-naive and -treated patients with metastatic castration-resistant prostate cancer (mCRPC), but also with a delay in time to development of Skeletal Related Events and in radiological skeletal progression. These bone benefits may be related to a direct effect on prostate cancer cells in bone or to a specific mechanism directed to bone microenvironment. To test this hypothesis we designed an *in vitro* study aimed to evaluate a potential direct effect of ABI on human primary osteoclasts/osteoblasts (OCLs/OBLs). We also assessed changes in bone turnover markers, serum carboxy-terminal collagen crosslinks (CTX) and alkaline phosphatase (ALP), in 49 mCRPC patients treated with ABI.

Our results showed that non-cytotoxic doses of ABI have a statistically significant inhibitory effect on OCL differentiation and activity inducing a down-modulation of OCL marker genes TRAP, cathepsin K and metalloproteinase-9. Furthermore ABI promoted OBL differentiation and bone matrix deposition up-regulating OBL specific genes, ALP and osteocalcin. Finally, we observed a significant decrease of serum CTX values and an increase of ALP in ABI-treated patients.

These findings suggest a novel biological mechanism of action of ABI consisting in a direct bone anabolic and anti-resorptive activity.

## INTRODUCTION

Several anticancer agents directly influence bone remodeling targeting specific bone cells such as osteoclasts (OCLs), osteoblasts (OBLs), osteocytes and, concomitantly, resulting in bone anabolic and anti-catabolic therapeutic effects. In this context, a number of bone-targeted agents are currently under preclinical and clinical investigation such as c-Src inhibitors, integrins inhibitors, cathepsin-K inhibitors, endothelin receptor antagonists and WNT signaling pathway modulators [1].

Abiraterone acetate (ABI) is a selective androgen biosynthesis inhibitor that potently and irreversibly blocks Cyp17 resulting in virtually undetectable serum and intratumoral androgen production in the adrenals, testes and prostate cancer cells [2, 3]. In phase III studies in metastatic castration-resistant prostate cancer (mCRPC) patients, it was demonstrated that ABI treatment is associated not only with a significant survival advantage in both chemotherapy-naive and chemotherapy-treated patients [4-6] but also, in docetaxel treated patients, with a better pain control from skeletal metastases, a delay in time to development SREs and in radiological skeletal progression. More specifically, 25% of patients developed a skeletal event in 9.9 months when treated with ABI and 4.9 months with placebo and time to first SRE was 25.0 months with ABI compared to 20.3 months with placebo [4, 5]. These ABI effects on metastatic bone disease may be secondary to a systemic control of the disease due to a direct antitumor effect that, in turns, leads to a decrease of cancer cells/OCLs/OBLs vicious circle or, alternatively, to a specific action directed to bone microenvironment. To test this second hypothesis we have designed a translational study aimed to investigate a potential bone direct effect of ABI in an *in vitro* model of human primary OCLs/OBLs and in a prospective cohort of castration resistant prostate cancer patients in which bone turnover markers were assessed during ABI treatment.

## RESULTS

### Cyp17a1 and androgen receptor expression during osteoclast and osteoblast differentiation

Cyp17a1 expression was evaluated at different stages of osteoclast/osteoblast differentiation by real-time PCR. Osteoclast mRNA levels were assessed at three different time-points during the differentiation protocol [day 0 (monocyte), day 6 (pre-osteoclast), day 12 (mature osteoclast)] as well as its expression in the osteoblasts was evaluated at day 0 (mesenchymal cells), day 14 (pre-osteoblast) and day 21 (mature osteoblast). Cyp17A1 was expressed during all phases of osteoclast/osteoblast maturation with a significant increase of mRNA levels at early stage of osteoclast differentiation (monocytes vs pre-OCL *p* < 0.0001; monocytes vs OCL *p* < 0.0001) (Fig. S1). Conversely Cyp17a1 mRNA levels remained unchanged during the osteoblast maturation process (Fig. S1). During OCLs/OBLs differentiation Androgen Receptor (AR) expression levels were also assessed because it was shown by others a direct activity of steroids on bone cells [7-12]. AR was expressed at different stages of osteoclast differentiation with a significant decrease in the levels of transcripts in the mature osteoclasts (monocytes vs OCL *p* < 0.001). Similarly to what observed for Cyp17a1, AR mRNA levels were stable during all the phases of OBLs differentiation (Fig. S1).

### Effect of ABI on primary osteoclast differentiation and activity in presence or absence of steroids

**Figure 1:**
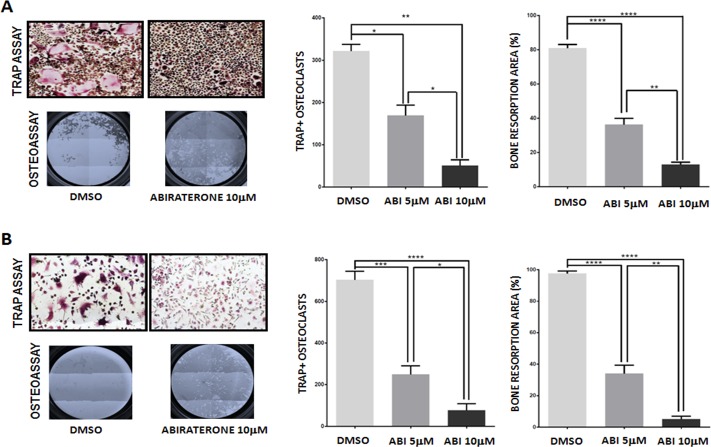
Effect of abiraterone treatment on primary osteoclast TRAP and Osteoassay in treated and untreated osteoclasts (DMSO) in presence (**A**) and absence (**B**) of steroids. *(*P* < 0.05) **(*P* < 0.001) ** (*P* < 0.0001) ****(*P* < 0.00001).

ABI was administered to primary cells at two different concentrations, 5μM and 10μM similarly to other preclinical studies that tested 10μM as maximum dose in *in vitro* assays [13-15]. Both doses did not impact on osteoclast viability excluding a possible cytotoxic effect (Fig. S3). ABI was added to osteoclast cell cultures every 3 days and osteoclast differentiation was evaluated at the end of the differentiation protocol (day 12) by functional TRAP assay. ABI treatment had a statistically significant inhibitory effect on osteoclast maturation reducing the number of mature TRAP+ osteoclasts compared with control (DMSO) (DMSO vs ABI 5 μM *p* < 0.05; DMSO vs ABI 10 μM *p* < 0.001; ABI 5 μM vs ABI 10 μM p = 0.032) (Fig. 1A). The effect of ABI treatment on osteoclastic activity was tested by seeding monocytes on wells coated with inorganic calcium phosphate to mimic bone matrix and evaluating the reabsorbed areas (pits) produced by osteoclasts at the end of the differentiation protocol. ABI significantly inhibited bone resorption interfering with osteoclast function (DMSO vs ABI 5 μM *p* < 0.0001; DMSO vs ABI 10 μM *p* < 0.0001; ABI 5 μM vs ABI 10 μM p = 0.020) (Fig. 1A). Moreover, the effect of ABI on osteoclast differentiation and activity was also assessed using a charcoal-treated serum to deplete the steroid content in the culture medium. In this deprivation status the rate of mature osteoclasts significantly increased as well as the ability of these cells to reabsorb bone matrix (*p* < 0.001) (Fig. S2). In such deprivation model, TRAP and resorption assay confirmed the anti-resorptive action of ABI suggesting an androgen-independent inhibitory mechanism (Fig. 1B).

### Effect of ABI on primary osteoblast differentiation and activity in presence or absence of steroids

**Figure 2:**
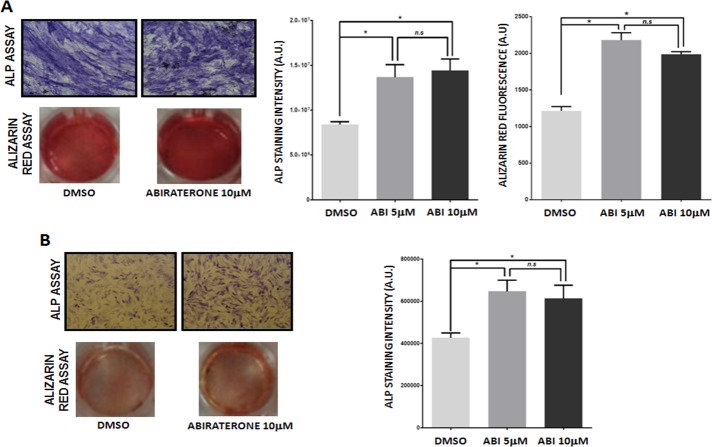
Effect of abiraterone treatment on primary osteoblast ALP and Alizarin Red assay in treated and untreated osteoclasts (DMSO) in presence (**A**) and absence (**B**) of steroids. *(*P* < 0.05).

The effect of ABI on osteoblastic differentiation has been evaluated in both presence and absence of steroids using ALP assay that allows identifying the expression of ALP, the main enzyme marker for osteoblasts. Following ABI treatment osteoblast cultured in steroid-containing medium showed a significant increase in the ALP positivity (DMSO vs ABI 5 μM p = 0.035; DMSO vs ABI 10 μM p = 0.30) (Fig. 2A). Osteoblast ability to produce bone matrix was analyzed only in presence of androgen since steroid deprivation made cells unable to induce calcium phosphate deposits. ABI increased significantly bone matrix deposition stained by Alizarin red assay (DMSO vs ABI 5 μM p = 0.026; DMSO vs ABI 10 μM p = 0.014) (Fig. 2A). These data demonstrated that, unlike osteoclasts, osteoblasts require a source of steroids to reach the complete differentiation, although ABI treatment results in a partial differentiation rescue in the steroid-depleted condition suggesting an androgen-independent anabolic effect of ABI. Indeed following ABI administration the percentage of ALP+ osteoblasts was significantly increased compared to control (DMSO *vs* ABI 5 μM p = 0.018; DMSO *vs* ABI 10 μM p = 0.020) as shown in Fig. 2B.

### Effect of ABI on osteoclast/osteoblast markers

**Figure 3:**
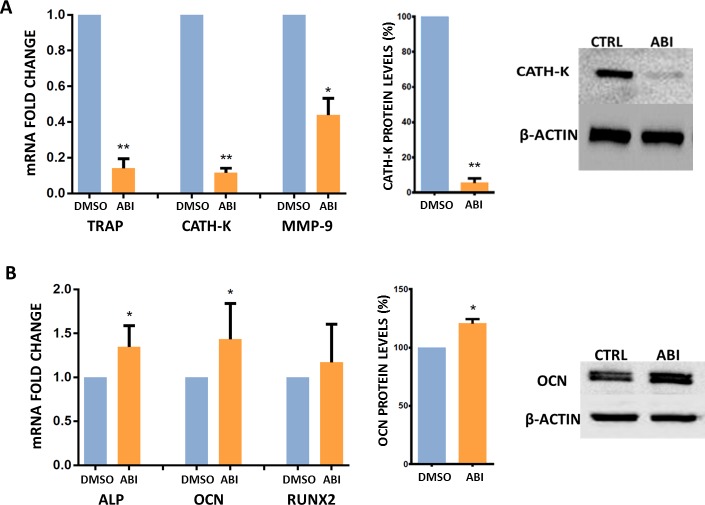
Gene and protein expression analyses (**A**). TRAP, CATH-K and MMP-9 mRNA levels (Real Time PCR) and CATH-K protein levels (Western Blot) in treated and untreated osteoclasts (DMSO) cultured with steroids. (**B)**. ALP, OCN, and RUNX2 mRNA levels (Real Time PCR) and OCN protein levels (Western Blot) in treated and untreated osteoclasts (DMSO) cultured with steroids. *(*P* < 0.05) **(*P* < 0.001).

Compared to baseline values, ABI significantly down-modulated osteoclast markers such as TRAP (*p* < 0.001), cathepsin-K (*P* < 0.001), MMP-9 (P = 0.015) and up-regulated the expression of osteoblast’ ALP (p = 0.015) and osteocalcin (p = 0.034) genes both in presence and absence of steroids (Fig. 3A and 3B). The reduction of cathepsin-K levels and the increase of osteocalcin expression were confirmed by Western Blot analyses (Fig. 3A and 3B).

### Effect of ABI on bone turnover markers in prostate cancer patients

Forty-nine consecutive patients were recruited. Eighteen of them have the last follow up visit 10 months after the end of treatment. Patients’ characteristics are reported in Table 1. Median age was 67 years. Sixteen of them had Gleason Score (GS)=7, six GS<7, twenty-three GS>7. 16 patients had bone metastases, 18 patients bone and lymph nodes metastases, 9 patients lymph nodes metastases, 3 patients bone and visceral metastases, 1 patient visceral metastases, 1 patient bone metastases and local recurrence, 1 patient bone, lymph nodes and visceral metastases. Twenty patients started zoledronic acid at least three months before beginning ABI treatment. The median number of zoledronic acid doses before ABI therapy was 12.

**Table 1:** Patients characteristics

Age (median)	67 years
Gleason score (GS): =7 <7 >7 Unknown	Patients 16 6 23 4
Metastasis: bone bone and lymph node lymph node bone and visceral visceral bone and local recurrence bone, visceral and lymph node	Patients 16 18 9 3 1 1 1
Zoledronic acid treatment during ABI	Patients 20
ECOG PS: 0 1 2	Patients 31 15 3

**Figure 4:**
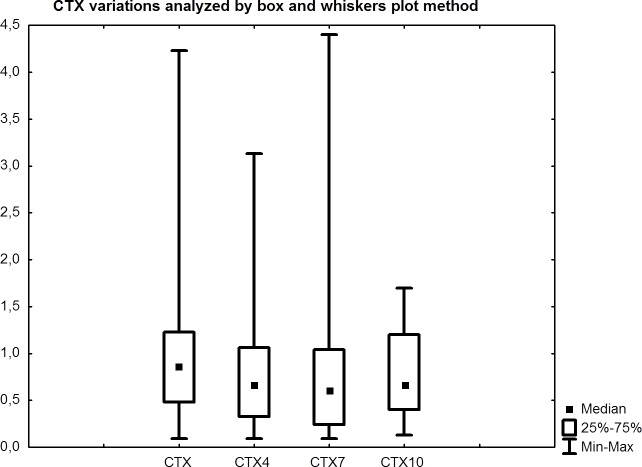
Comparison between CTX at baseline and after three, six and nine months

A significant decrease in CTX values was observed: at baseline the median value was 0.86 ng/mL (95% confidence interval [CI]: 0.84-1.25), and at three, six and nine months it was 0.78 ng/mL (95%CI: 0.67-1.01) (p = 0.077), 0.61 ng/mL (95%CI: 0.73-1.19) (p = 0.027) and 0.66 ng/mL (95%CI: 0.38-0.71) (p = 0.006), respectively (Table 2, Fig. 4). Compared to median value at baseline a CTX decrease by 9.3%, 29% and 23% was observed at three, six and nine months, respectively.

**Table 2:** Difference in median level of bone resorption and formation markers

CTX	Baseline ng/mL	Three months ng/mL	Six months ng/mL	Nine months ng/mL
Median, 95% IC	0.86, (0.84-1.25)	0.78, ( 0.67-1.01)	0.61, (0.73-1.19)	0.66, (0.38-0.71)
p (compare to baseline)		p=0.077	p=0.027	p=0.006
ALP	Baseline U/L	Three months U/L	Six months U/L	Nine months >U/L
Median, 95% IC	123, (126-261)	143, (255-382)	126, (200-327)	190, (172-344)
p (compare to baseline)		p=0.01	p=0.62	p=0.28

**Figure 5:**
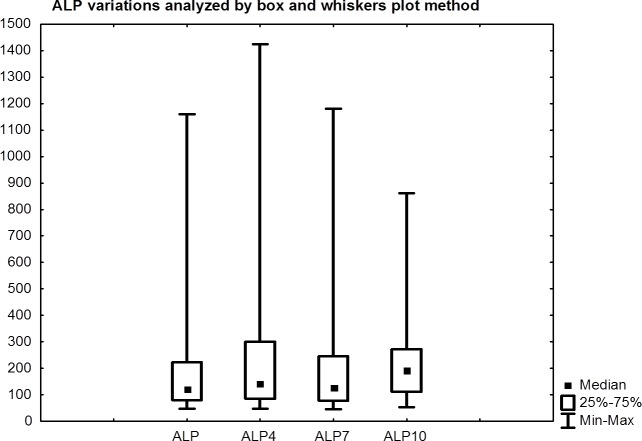
Comparison between ALP at baseline and after three, six and nine months

ALP did not show a significant increase, median 123 U/L (95%CI: 126-261) and 190 U/L (95%CI 172-344) at baseline and nine months (p = 0.28), respectively. However, the comparison between ALP at baseline and after 3 months showed a significant increase (p = 0.010) (Table 2, Fig. 5). Compared to median value at baseline an ALP increase by 16.3%, 2.4% and 54.5% was observed at three, six and nine months, respectively.

## DISCUSSION

The results of the present study demonstrate for the first time a direct bone anabolic and an anti-resorptive effect of ABI both *in vitro* and in castration resistant prostate cancer patients. Indeed, ABI is able to specifically modulate osteoclast/osteoblast differentiation without generating any cytotoxic and/or proliferative effect at the administrated doses. Moreover, our findings show that ABI regulates gene expression in bone cells modulating mRNA levels of key osteoclastic/osteoblastic genes. Intriguingly, we observed that anabolic and anti-resorptive effects are produced both in presence and absence of steroids suggesting a non-canonical mechanism of action that seems to be, at least in part, androgen-independent.

Recently Bruno et al. reported about an alternative mechanism analyzing the effect of ABI and other specifc CyP17 inhibitors in an androgen-independent prostate cancer cell line (PC-3) [16]. The treatment induced a rapid release of calcium from the endoplasmic reticulum (ER) resulting in disruption of calcium homeostasis. This, in turns, led to the ER stress response (ERSR), also known as “unfolded protein response” (UPR) through the phosphorylation of the translation elongation factor eIF2α and the up-regulation of stress response genes, triggering cell cycle arrest. Moreover, recent evidences showed that eIF2α phosphorylation promotes the activation of ATF4, a key osteoblastic transcriptional factor, leading to osteocalcin up-regulation and down-modulation of a key pro-osteoclastic factor, NFATc1, resulting in bone matrix production [17]. Taken together these data suggest a novel potential molecular mechanism of ABI that, acting through eIF2α phosphorylation, may promote osteoblastogenesis and inhibit osteoclast-dependent bone resorption. These promising *in vitro* results provided a strong rationale to design a translational study to investigate the potential modulation of serum bone turnover markers by ABI treatment in a cohort of 49 metastatic castration resistant prostate cancer patients (mCRPC). Our findings showed that in these patients ABI significantly inhibited bone resorption as documented by a decrease of serum CTX values and enhanced new bone formation as shown by an increase of ALP in agreement with *in vitro* data.

Overall, our pre-clinical and clinical data provide a biological rationale for the high efficacy of ABI treatment in improving multiple skeletal disease specific clinical endpoints as recently documented in the context of phase III clinical trials. Molecules that simultaneous target prostate cancer cells and bone microenvironment could significantly influence future therapeutic approaches in order to achieve a better disease control and management of prostate cancer bone metastases. If further prospective confirmation will be obtained these pivotal results justify a potential synergistic effect of ABI with bone-targeted therapies (bisphosphonates and denosumab).

In conclusion, these findings represent the first evidence of a novel mechanism of ABI directed on bone microenvironment together with the known antitumoral effect on CRPC cells and pave the way to new treatment scenarios for bone metastatic prostate cancer.

## MATERIALS AND METHODS

### Primary cell culture

Human peripheral blood mononuclear cells (PBMCs) were isolated from buffy-coat of 10 male healthy donors by Lympholyte®-H density gradient (Cedarlane Laboratories) and monocytes were sorted using beads conjugated anti-human CD14 (Miltenyi Biotech) and cultured for 12 days in RPMI culture medium (Euroclone) supplemented with 10% fetal bovine serum (Hyclone, Thermo Scientific) or 10% charcoal stripped serum (Sigma-Aldrich), 100 units/ml penicillin, 100 mg/ml streptomycin (Euroclone), 2 mM L-glutamine (Euroclone), 25 ng/mL macrophage-colony stimulating factor (M-CSF) and 50 ng/mL receptor activator of nuclear factor kappa-B ligand (RANKL) (R&D Systems) to differentiate them into osteoclast. During the differentiation protocol (from day 1 to 12), cells were treated with 5-10 μM of ABI acetate (Selleckem) or vehicle as control.

Primary human osteoblasts were differentiated from human mesenchymal stem cells (hMSCs) gently gifted by Cell Culture Laboratory, Department of Biomedicine and Prevention of the University Tor Vergata, Rome. HMSCs were cultured for 21 days in alpha MEM (Euroclone) supplemented with 15% fetal bovine serum or 15% charcoal stripped serum, 100 units/ml penicillin, 100 mg/ml streptomycin, 2 mM L-glutamine, 10 mM beta-glycerophosphate (Sigma-Aldrich), 50 μM ascorbic acid (Sigma-Aldrich) and 100 nM dexamethasone (Sigma-Aldrich). During the differentiation protocol (from day 1 to 21), cells were treated with 5-10 μM of ABI acetate or vehicle as control.

All culture media, growth factors, cytokine and ABI acetate were replaced every 3-4 days [18-19].

### Osteoclast functional assays

At the end of the differentiation protocol (day 12) culture medium was removed and cells were fixed with 4% formaldehyde for 5 minute and stained with leukocyte acid phosphatase (TRAP) kit (Sigma-Aldrich) according to manufacturer's instructions. Stained positive cells (>3 nuclei) were then counted [20].

Osteoclasts activity was assessed culturing cells on plates coated with a synthetic inorganic bone mimetic matrix (Osteoassay, Corning). At day 12 the culture medium was removed and plates were filled with sodium hypochlorite solution to evaluate the ability of mature osteoclasts to reabsorb this substrate; pits produced by osteoclasts re-absorptive activity were quantified by ImageJ software.

### Osteoblast functional assays

On day 21 cells were fixed with 4% formaldehyde for 5 minute and stained with alkaline phosphatase (ALP) kit (Sigma-Aldrich) according to the manufacturer's protocol. ALP positivity was quantified by ImageJ software.

In order to detect bone matrix deposition as a marker of osteoblastic activity cells were fixed with 4% formaldehyde for 20 minute and stained with alizarin red for 1 hour at room temperature. Alizarin red fluorescence was detected at 470 nm and quantified by spectrofluorimeter (Tecan Infinite M200Pro) [19].

### MTT assay

Cell viability was evaluated by cell growth determination kit, MTT-based assay (Sigma-Aldrich), and performed according to manufacturer's instructions; the optical density (OD) of the colored complex formed was read by spectrophotometer with 570 nm wavelength and background absorbance at 690 nm wavelength was subtracted.

### RNA extraction and gene expression analysis

Total RNA was extracted from osteoclast and osteoblast cells at the end of the differentiation protocol using the Trizol reagent (Invitrogen) according to the manufacturer's instructions. RNA was treated with DNase buffer and DNase (DNAse Turbo, Applied Biosystems) to avoid genomic DNA contamination. cDNA was produced using the High Capacity cDNA Reverse Transcription kit (Applied Biosystems) according to the manufacturer's instructions. mRNA levels were measured by quantitative real-time polymerase chain reaction (qRT-PCR) using TaqMan Gene Expression Assays in 7900HT Real-Time PCR System (Applied Biosystems). *Tartrate resistant acid phosphatase (TRAP)* (Hs00356261_m1), *cathepsin-K* (Hs00166156_m1), *metalloproteinase-9* (Hs00234579_m1), *alkaline phosphatase (ALP)* (Hs01029144_m1), *osteocalcin* (Hs00234160_m1) and *runt-related transcription factor 2 (RUNX2)* (Hs00231692_m1) expression levels were normalized to the endogenous housekeeping gene *glucuronidase beta (GUSb)* (Hs99999908_m1) in both untreated and treated samples using the ΔCT calculation. Subsequently relative expression levels in treated samples were normalized to the mRNA levels detected in control samples using the ΔΔCT calculation [21].

### Protein extraction and western blot analysis

Cell lysates was obtained using radioimmunoprecipitation assay buffer (RIPA buffer) (Sigma-Aldrich) and quantified using DC protein assay kit (Bio-Rad). Twenty mg of the total protein extract from each sample was loaded on 8%/15% SDS-PAGE gels, transferred onto nitrocellulose membranes through Trans-Blot Turbo Transfer System (Bio-Rad) and incubated in a blocking buffer (TBST 1X with 5% non-fat dry milk) for one hour. Mouse monoclonal anti-human Cat-K (Santa Cruz Biotechnologies), rabbit polyclonal anti-human OCN (Santa Cruz Biotechnologies) and mouse anti-human Actin-β (Sigma-Aldrich) were incubated for 2 hours at room temperature. Anti-rabbit/mouse HRT-coniugated antibody (Abcam) was used and the chemiluminescence signal detected using ChemiDoc (Bio-Rad) and Quantity One software (Bio-Rad) to quantify the bands’ signal intensity.

### Patients

Patients with metastatic castration resistant prostate cancer (mCRPC) with or without bone metastases in clinical progression following treatment with docetaxel and enrolled in the ABI acetate expanded access program at the Division of Medical Oncology-S. Luigi Hospital, Orbassano were prospectively assessed. ABI acetate was administered at the dose of 1.000 mg daily with prednisone 5 mg twice a day in combination with luteinizing hormone releasing hormone analogue until radiological or serological progression. A subgroup of patients was also treated with zoledronic acid at standard unchanged doses in the last three months before starting ABI. All patients were evaluated for bone formation and resorption.

Serum alkaline phosphatase (ALP) as a marker of osteoblast activity and serum c-telopetide of type-I collagen (CTX) as a marker of bone resorption were assessed every 3 months at a centralized laboratory at San Luigi Hospital Orbassano. Total ALP was evaluated by standard automated analytical procedures (Architect, Abbott) and normal levels ranged between 30 and 120 U/L. Serum CTX level was measured using a commercial ELISA kit (IDS-iSYS CTX-I, Immunodiagnostic Systems Ltd). Normal values, minimum detectable concentrations, intra- and inter assay coefficients of variation of CTX were as follows: 0.12-0.75 ng/mL, 0.023 ng/mL, 3.2 and 6.3 %.

### Statistical analysis

*In vitro* data were analyzed using the Student t test and One-Way ANOVA test followed by Tukey's multiple comparison tests. The graphics processing and statistical tests were performed using the program GraphPad Prism (San Diego, CA).

For marker analyses Wilcoxon's matched pairs sign-rank test was used to compare pair data at baseline and after 3, 6 and 9 months of treatment. All *p*-values reported were two-sided; *p*-values <0.05 were chosen for statistical significance. Statistical computation was performed using the SPSS for Windows software package.

## SUPPLEMENTARY MATERIAL FIGURES


