# Supplementary material for: Biological and clinical effects of abiraterone on anti-resorptive and anabolic activity in bone microenvironment
Source: Oncotarget. 2015 Mar 30;6(14):12520–8. doi: 10.18632/oncotarget.3724 (PMC4494955; doi:10.18632/oncotarget.3724)
Supplement: Supplementary file 1 [file oncotarget-06-12520-s001.pdf]

Biological and clinical effects of abiraterone on anti-resorptive and anabolic activity in bone microenvironment

Supplementary Material

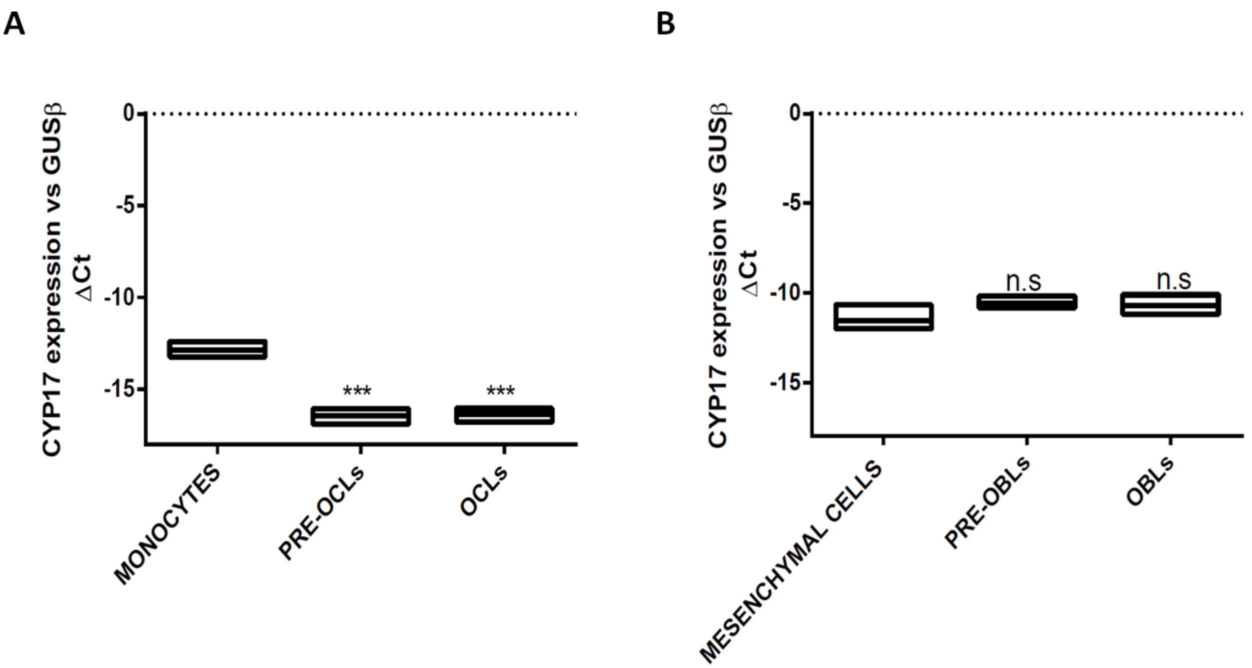

Fig. S1: CYP17A1 mRNA levels expressed during osteoclast (A) and osteoblast (B) differentiation normalized for GUSβ. * ($P<0.05$) ** ($P<0.001$) *** ($P<0.0001$) **** ($P<0.00001$)

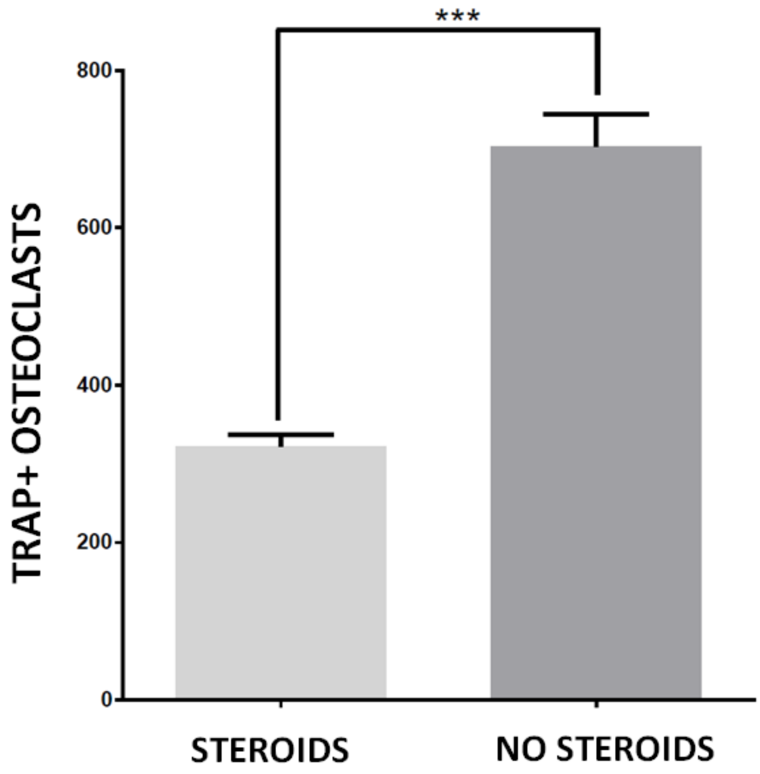

Fig. S2: Comparison of osteoclasts differentiation in presence/absence of steroids (Trap assay). * (P<0.05) ** (P<0.001) *** (P<0.0001) **** (P<0.00001)

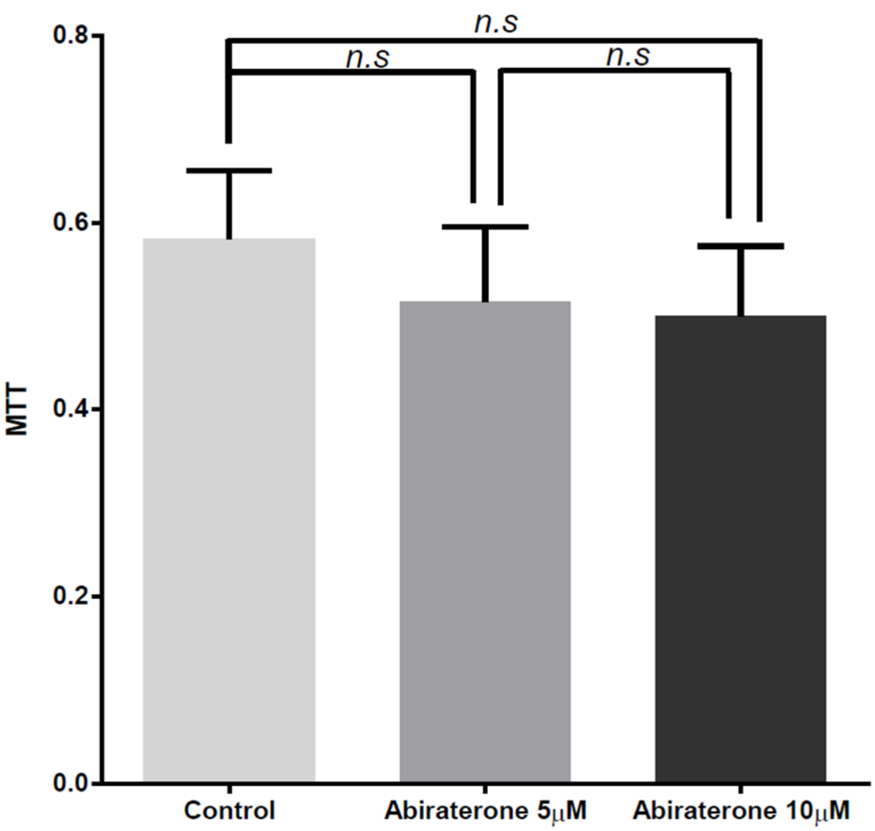

Fig. S3: Osteoclast Viability (MTT assay) * (P<0.05) ** (P<0.001) *** (P<0.0001) **** (P<0.00001)
